# Ablation of NPFFR2 in Mice Reduces Response to Single Prolonged Stress Model

**DOI:** 10.3390/cells9112479

**Published:** 2020-11-14

**Authors:** Ya-Tin Lin, Yi-Ling Huang, Sze-Chi Tsai, Jin-Chung Chen

**Affiliations:** 1 Department of Physiology and Pharmacology, Graduate Institute of Biomedical Sciences, Chang Gung University, Taoyuan 33302, Taiwan; yatin@mail.cgu.edu.tw (Y.-T.L.); m0701308@cgu.edu.tw (Y.-L.H.); 2 Healthy Aging Research Center, Chang Gung University, Taoyuan 33302, Taiwan; 3 Department of Biomedical Sciences, Chang Gung University, Taoyuan 33302, Taiwan; mandy85917@gm.ym.edu.tw; 4 Neuroscience Research Center, Chang Gung Memorial Hospital, Taoyuan 33305, Taiwan

**Keywords:** neuropeptide FF, NPFFR2, stress, anxiety, HPA axis, SPS

## Abstract

Mental stress is highly related to many clinical symptoms and disorders, as it activates the hypothalamic-pituitary-adrenocortical (HPA) axis to affect a wide variety of physiological functions. Furthermore, stress leads to the aberrations in HPA axis activity and disruptions of body homeostasis. It was previously shown that neuropeptide FF (NPFF) regulates the HPA axis through the activation of hypothalamus paraventricular nucleus (PVN), and genetic overexpression or pharmacological stimulation of NPFF receptor 2 (NPFFR2) triggers hyperactivity of HPA axis and suppresses behavioral correlates of emotion in mice. In this study, we further examined the role of NPFFR2 in stress response in mice by utilizing a single prolonged stress (SPS). SPS is considered a model of post-traumatic stress disorder (PTSD), and mice undergo physical restraint, forced swimming, and ether anesthesia within a day followed by social isolation for one week. NPFFR2 knockout B6 mice were generated by CRISPR/Cas9 technology and exposed to SPS. The NPFFR2 knockouts showed resistance to stress exposure-induced anxiety-like behaviors and HPA axis hyperactivity. Additionally, the hippocampal mRNA levels of glucocorticoid receptor and mineralocorticoid receptor were reduced in wild-type (WT) mice but not in NPFFR2 knockouts after stress exposure. Our data also suggested that NPFFR2 knockout mice have stronger negative feedback on the HPA axis after exposure to SPS. Mice with intra-PVN *Npffr2* shRNA injection displayed trends toward resistance to SPS exposure in both behavioral and molecular assays. Together, our findings suggest that NPFFR2 may be a potential therapeutic target for disorders relating to stress/anxiety and HPA dysregulation.

## 1. Introduction

Mental stress is an important factor that leads to different pathological symptoms or diseases, including psychological disorders, cardiovascular disease, immune system disorders, and even cancer [1]. Stress is widely known to activate the endocrine response system, especially the hypothalamic-pituitary-adrenocortical axis (HPA axis), which releases glucocorticoid (corticosterone [CORT] in rodent, or cortisol in human) to regulate various physiological processes [1,2]. Dysregulation of the HPA axis disrupts emotional regulation (leading to anxiety or depression), eating behavior, cognitive function, circadian rhythm, and the immune system [3]. Chronic stress is even more harmful than short-term stress, as it can interfere with neuroplasticity of the limbic system at both structural and functional levels, triggering other forms of aberrant HPA axis activity [4].

The hypothalamic paraventricular nucleus (PVN) is upstream of the HPA axis, and during stressful events, corticotropin-releasing hormone (CRH) is released from the PVN to evoke the secretion of adrenocorticotrophic hormone (ACTH) from the anterior pituitary; consequently, secretion of glucocorticoid from the adrenal cortex is stimulated [2]. HPA axis functions are largely controlled by two receptors, the mineralocorticoid receptor (MR) and the glucocorticoid receptor (GR), which bind to brain glucocorticoid with different affinities. MR is thought to maintain basal activity of HPA axis, and GR, which exhibits lower binding affinity, is activated predominantly by stress-induced, high-frequency, high-amplitude bursts of glucocorticoid secretion [5].

Neuropeptide FF (NPFF, FLFQPQRF-NH2) was first isolated from bovine brain and is known to regulate the pharmacological response to morphine, pain sensation, food intake, and mood disorders [6,7,8,9]. In the past decade, the function of NPFF in hypothalamus has been studied, with most of the focus on its regulation of the neuroendocrine system [10,11]. It has been shown that NPFF disinhibits GABAergic projections to the parvocellular PVN, which leads to the activation of downstream neural circuits [12]. One of its major receptors, NPFF receptor 2 (NPFFR2), is expressed in various sub-regions of the hypothalamus, including the PVN [13]. Correspondingly, pharmacological activation of NPFFR2 directly stimulates the HPA axis through the PVN, which increases c-Fos protein expression in the PVN, causing downstream CORT secretion and anxiety-like behaviors [14]. Both NPFFR2-overexpressing mice and mice chronically administered with NPFFR2 agonist exhibit depressive-like behaviors and biochemical changes that are similar to mice exposed to chronic stress [15]. Moreover, chronic stress leads to long-lasting activation of the HPA axis, thereby reducing hippocampal GR protein and impairing negative feedback on the HPA axis [15].

Because of its influence on HPA activity, NPFFR2 might be a potential therapeutic target for mood disorders. Single prolonged stress (SPS) is considered to be a model of post-traumatic stress disorder (PTSD) with aberrant HPA axis activity. In the current study, we investigated the impact of NPFFR2 signals in mice exposed to SPS. To test the influence of hypothalamic NPFFR2 on outcomes of SPS model, the NPFFR2 knockout (KO) and knockdown mice were used. NPFFR2 congenital KO mice were generated by CRISPR/Cas9 and intra-hypothalamic PVN Lenti-*Npffr2*-shRNA injection was utilized for this purpose.

## 2. Materials and Methods

### 2.1. Experimental Animals

Male and female C57BL/6 wild-type (WT) mice or NPFFR2 KO mice (8–12 weeks old, 24–30 g) were bred in an SPF environment at Chang Gung University (AAALAC accreditation, November 2018), maintained at 22 ± 1 °C, 50 ± 5% humidity, and 12 h light/dark cycle (lights on, 07:00). Mice were housed four or five per cage, after PCR genotyping at 3 weeks old. Food and water were available *ad libitum*. Animal handling and drug treatments were performed in strict accordance with the NIH Guide for the Care and Use of Laboratory Animals and approved by the IACUC at Chang Gung University (CGU 14-014). 

### 2.2. Generation of NPFFR2 KO Mice Using CRISPR/Cas9

Cas9 protein and in vitro transcribed single guide (sgRNA) targeting *Npffr2* (sgRNA1: GCAATGATACAGCATCACTGG; sgRNA2: GATCTTTGTCTTGTGCATGGTGG; sgRNA3: CCTTGCCATAAGTGATTTACTGG; sgRNA4: CCAGTAAATCACTTATGGCAAGG; Accession numbers: NM_133192) were purchased from BIOTOOLS Co., Ltd. (Taipei, Taiwan). The efficiencies of four sgRNA were tested by in vitro by incubating Cas9 protein with a PCR amplicon covering the mouse NPFFR2 locus (forward, 5′ CTC CTT TGT TAA GGT CCA CCA 3′; reverse, 5′ TGG AAC ACT TCT GGG ACC TC 3′; PCR product 467 bp). 

C57BL6/JNarl and ICR mice were purchased from the National Laboratory Animal Center (Taipei, Taiwan) and were used as embryo donors and foster mothers, respectively. Female C57BL6/JNarl mice (7–8 weeks old) were super-ovulated by intraperitoneal (i.p.) injection of 7.5 international unit (IU) of pregnant mare serum gonadotropin (PMSG, Sigma-Aldrich, St. Louis, MO, USA) and 10 IU of human chorionic gonadotropin (hCG, Sigma-Aldrich) 48 h after PMSG injection. The super-ovulated female mice were mated with male mice, and the resultant fertilized embryos were collected from the oviducts. Recombinant Cas9 protein/sgRNA2 and Cas9 protein/sgRNA3 complex (Cas9 protein 200 ng/μL: sgRNA 50 ng/μL) were then microinjected into oocyte pronuclei harvested from super-ovulated female mice. The manipulated embryos were then implanted to the oviducts of pseudo-pregnant foster mothers (ICR mice) to produce live animals. Twenty-nine newborn mice were generated. After PCR and sequencing selection, four NPFFR2 KO F0 mice were selected and designated as number 1, 7, 24, and 29. Male and female heterozygous mice were mated to produce the homozygous NPFFR2 KO mice and sex comparable littermate WT mice.

NPFFR2 deletion was verified by PCR of genomic DNA and cDNA, with reverse primers designed to target the location of the deletion segment. The PCR primer sequences are as follows: forward, 5′ ACT ATC TCC ACC AGC CCC AA 3′; reverse, 5′ GTT GTC CAG CAA TGT GAT AGG C 3′; PCR product 209 bp for WT mice. The PCR primer sequences for genotyping are as follows: forward, 5′ CTC CTT TGT TAA GGT CCA CCA 3′; reverse, 5′ TGG AAC ACT TCT GGG ACC TC 3′; PCR product 467 bp for WT mice and 388 bp for No. 29 NPFFR2 KO mice.

### 2.3. Behavioral Tests

Three different sets of male mice were used to measure the basal behavioral difference between WT and NPFFR2 KO mice. One set of mice was monitored for the growth curve (WT, *n* = 11; KO, *n* = 7). Another set of mice was used for the water maze learning test (WT, *n* = 4; KO, *n* = 5). Last set of mice was used for a panel of behavioral tests with a least 3 days interval between each test (WT, *n* = 9; KO, *n* = 10). In addition, one set of female mice was used to measure the basal behavioral difference between WT and NPFFR2 KO mice with a least 3 days interval between each test (*n* = 5 vs. *n* = 5).

#### 2.3.1. Locomotor Activity

Mice were adapted in the testing room for 2 h before the test starts. Locomotor activity was monitored in an open field arena (40 × 40 cm^2^) with a 30-cm high opaque white wall. Mice were put into the corner of the arena and faced to the wall. Body movement within 60 min was recorded by video and tracked by the EthoVision tracking system (Noldus, Wageningen, The Netherlands). The locomotor activity is presented as the distance that mice moved (cm) in every 5 min bin, for a total session of 60 min. 

#### 2.3.2. Prepulse Inhibition (PPI)

PPI test was performed in a startle chamber acquired from San Diego Instruments (SR LAB^TM^, SD Instruments, San Diego, CA, USA). Mouse was left in a plexiglas cylinder for 5 min (with 3.8 cm inside diameter), which was attached to a vibration-detecting platform. A 65 dB background sound was applied during the test. Vibration signals were recorded after mice were stimulated with 40 ms of 120 dB acoustic pulse with or without prepulse. The 120 dB acoustic pulse was in combination with 20 ms of three random prepulses (pp): 69 dB, 73 dB, and 80 dB, with a 100 ms interval between pulse and prepulse stimulation. Five different stimulations were randomly executed for 10 times, including no stimulation, p120 dB, pp69 dB-p120 dB, pp73 dB-p120 dB, and pp80 dB-p120 dB. A random interval of 5–30 s was applied between stimulations. The average of vibration signals were recorded and calculated as follows: PPI% = (pulse-prepulse)/pulse × 100%.

#### 2.3.3. Sucrose Preference Test

Mouse was singly housed in a testing cage with food and water for adaptation, 24 h prior to the test day. Afterward, two 400 mL bottles, containing tap water or 1% sucrose solution, were supplied overnight for 18 h. Overnight consumption (18:00 to 12:00 am) from each bottle was calculated by weighing the bottles (g). Data are presented as percentage of sucrose intake/(sucrose + water intake).

#### 2.3.4. Water Maze

Spatial learning was assessed by the Morris water maze. The maze was a circular pool with 120 cm diameter and 40 cm height and filled with 25 °C ± 2 °C water. The water was made opaque by adding non-fat milk and was separated into four equal quadrants. There was a hidden square platform (10 cm in diameter) which was located 1 cm below the water surface in the center of quadrant four. In the acquisition trails, a mouse was put into the tank to allow to find a hidden platform within 120 s (one trial). Four trials per day were performed for four days in total, and the latency for mice to find the platform was recorded. Each mouse was allowed to stand on the platform for 30 s at the end of each trail. In each day, mouse started the four trials from four different quadrants with 1–2 h interval between each trial. The mouse body tracks were recorded by EthoVision tracking system.

#### 2.3.5. Forced Swimming Test (FST)

Mice were adapted in the testing room for 2 h before the test starts. Mice were placed in a water cylinder with 50 cm depth and 15 cm diameter, which was filled to 30 cm deep with tap water at 25 ± 2 °C. Immobility time during the 6 min testing session was calculated from a video replay and was recorded in second.

#### 2.3.6. Tail Suspension Test (TST)

Mice were adapted in the testing room for 2 h before the test starts. Mice were suspended from a shelf 50 cm high and 10 cm from the back wall by securing the tail to the edge of the shelf. The immobility time during the 6 min testing session was calculated from a video replay and was recorded in second.

#### 2.3.7. Novelty-Suppressed Feeding (NSF)

Mice were fasted for 24 h before the test without limiting water intake. On the next day, mice were transferred to a new cage and acclimatized for 1 h. The original cage was designated as the home cage. During the test, mice were transferred to a black box (40 × 40 cm^2^) with a piece of white paper (10 × 10 cm^2^) placed in the center of the box, which contained a new food pellet in the center. Each mouse was placed in the same corner of the chamber to start the test. The latency with which mice first bit the food pellet was recorded with a 6 min cut-off time. Afterward, mice were moved back to the home cage and the amount of food consumption in the home cage was recorded for 5 min by measuring the change of weight for the food pellets (g).

#### 2.3.8. Light–Dark Box

The light-dark box contains a light chamber (white, 15 × 20 × 20 cm^3^) and a covered dark chamber (black, 30 × 20 × 20 cm^3^) with an open gate (4 × 4 × 1 cm^3^) between the two chambers. Mice were placed in the dark chamber, faced back to the gate and permitted to move freely between the two chambers for 10 min. The duration mice spent in each chamber was calculated from a video replay.

#### 2.3.9. Open Field Test (OFT)

The OFT was performed in an open field arena (40 × 40 cm^2^), with the same chamber used to test the locomotor activity. The outer zone was defined as an 8-cm wide strip around the outer edge of the arena. The rest of the area (24 × 24 cm^2^) was defined as the inner zone. The mouse body tracks were recorded by EthoVision tracking system. The durations mice spent in the inner and outer zones were calculated within 10 min of test.

#### 2.3.10. Elevated Plus Maze (EPM)

The EPM consists of two open arms and two closed arms, elevated 100 cm off the ground. The length of each arm is 30 cm, and the width is 5 cm. The closed arms have an additional black wall (20 cm high) on both sides and at the end of the arm. A mouse was placed on the middle of the elevated maze, faced toward an open arm, and its body track was video recorded for a 6-min session. The durations that the mouse spent in open and closed arms and the numbers of entries in open arms were calculated from the video replay. Mice were considered to have entered an arm once the middle part of their body passed the border of the arms.

### 2.4. Single Prolonged Stress (SPS)

Mice were randomly assigned to control or SPS groups. To perform SPS, male mice were first physically restrained for 2 h and then immediately put into the water cylinder (the same cylinder used for the FST) for 20 min. After mice recuperated from forced swimming for 15 min, mice were exposed to ether for 2 min and then put back into a new cage for single housing.

On the afternoon of day 7, facial blood was first collected from the mice at 12:00–14:00, and mice were then subjected to a sucrose preference test (from 16:00 to 10:00 on day 8). EPM was performed at 12:00–18:00 on day 8. OFT was performed at 12:00–14:00 on day 9. FST or TST was performed at 16:00–18:00 on day 9. Mice were then sacrificed to collect the brain tissues on day 10. For control group, mice were housed 4 to 5 per cage, and were gently stroked for few minutes every 3 days to avoid the anxious behavior while performing the behavioral tests.

Three different sets of male WT and NPFFR2 KO mice with *n* = 4–5 per group were exposed to SPS (including WT-control, WT-SPS, NPFFR2 KO-control, and NPFFR2 KO-SPS groups). The behavioral difference and serum CORT were validated in the first and second sets of mice and the data were pooled. Of which, FST was only performed on the first set of mice, and TST was only performed on the second set of mice. In the third set of mice, dexamethasone suppression test (DST) was performed on day 8 after SPS exposure without the behavioral test. Result from one of the NPFFR2 KO mouse exposed to SPS in the EPM test was excluded because the mouse fell from an open arm platform during the test (no injury was identified). Another set of male WT mice (*n* = 5 per group) was exposed to SPS after recovered from intra-PVN injection of control or *Npffr2*-shRNA.

### 2.5. RNA Extraction and Real-Time PCR

Total RNA was isolated using TRIzol^®^ reagent (Invitrogen, Carlsbad, CA, USA). cDNA was made using Superscript III reverse transcriptase (Invitrogen). Gene expression was measured by real-time PCR, using SYBR (Bio-Rad, Hercules, CA, USA) and CFX96 real-time PCR detection system (Bio-Rad). The PCR protocol was: 95 °C, 10 min, followed by 95 °C, 15 s and 60 °C, 30 s for 40 cycles. Rpl35a served as an internal control. The primer sequences are as follows: *Nr3c1* (GR)-forward: 5′AGC TCC CCC TGG TAG AGA C 3′; *Nr3c1* (GR)-reverse: 5′ GGT GAA GAC GCA GAA ACC TT 3′; *Gilz*-forward: 5′AAC ACC GAA ATG TAT CAG ACC C 3′; *Gilz*-reverse: 5′GTT TAA CGG AAA CCA CAT CCC CT 3′; *Fkbp5*-forward: 5′GGG TGT ACG CCA ACA TGT TC 3′; *Fkbp5*-reverse: 5′GAG GAG GGC CGA GTT CAT T 3′; *Nr3C2* (MR)-forward: 5′CCA GAA AAC GTG TCA AGC TCT 3′; *Nr3C2* (MR)-reverse: 5′GTT GTC CTT CCA CGG CTC TT 3′; *Rpl35a*-forward: 5′GCT GTG GTG CAA GGC CAT TTT 3′; *Rpl35a*-reverse: 5′CCG AGT TAC CTT TCC CCA GAT CAC 3′.

### 2.6. CORT ELISA

After collecting facial blood, the blood was clotted at room temperature for 30 min and centrifuged at 13,000 rpm for 15 min to collect the supernatant. Serum CORT was analyzed with a commercial ELISA kit (Enzo Life Sciences, Farmingdale, NY, USA), according to the manufacturer’s protocols. The SPS-induced serum CORT levels were calculated according to the following: levels of serum CORT after SPS exposure/the average level of serum CORT in corresponding control group.

### 2.7. Dexamethasone Suppression Test (DST)

Mice were i.p. injected with 0.1 mg/kg dexamethasone (Sigma, St. Louis, MO, USA) 1 h before mice were physically restrained for 10 min in a mouse restrainer. Facial blood was collected immediately after the restraint, under anesthetization with 2% isoflurane. Samples were analyzed by the CORT ELISA kit.

### 2.8. Intra-PVN Lenti-Npffr2-shRNA Delivery

WT male mice were anesthetized by i.p. injection of ketamine (67 mg/kg) and xylazine (34 mg/kg), and then secured in a stereotaxic instrument (David Kopf Instruments, Tujunga, CA, USA). *Npffr2*-shRNA was delivered into each (bilateral) hypothalamic PVN by a micro-syringe pump at a flow rate of 1 μL/min for 1 min, as previously described [15]. The injection coordinates for PVN were: AP, −0.94 mm; L, ± 0.25 mm and DV, −4.8 mm to bregma [16]. Mice were injected with 4 mg/kg ampicillin and 5 mg/kg meloxicam to prevent inflammation and provide pain relief. SPS was performed 1 to 2 weeks after mice recovered from surgery.

### 2.9. Statistical Analysis

All data are expressed as mean ± standard error mean (SEM). Statistical analyses are performed using Prism7 (GraphPad, San Diego, CA, USA) and analyzed by unpaired Student’s *t*-test or two-way ANOVA with post-hoc Bonferroni’s multiple comparison test. The specific analysis methods were mentioned in the results section and the figure legends. *p*-values below 0.05 were considered statistically significant. The result of two-way ANOVA was presented as F ratio (df of effect A, df of effect B). df = degree of freedom.

## 3. Results

**Figure 1:**
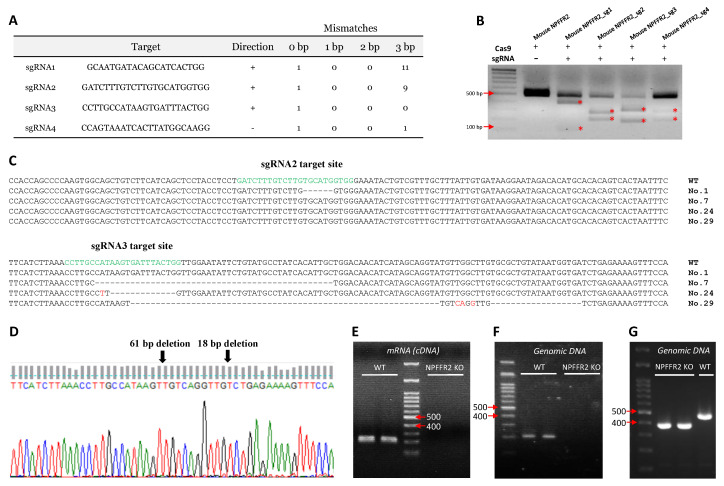
Generation of neuropeptide FF receptor 2 (NPFFR2) knockout (KO) mice. NPFFR2 KO mice were generated by CRISPR/Cas9 technology. (**A**) The sequences of four tested sgRNAs. (**B**) The gene editing efficiencies of sgRNAs were tested by incubating with Cas9 protein and PCR amplicons of the mouse NPFFR2 locus. *, indicates the digested segments. (**C**) DNA sequences of NPFFR2 KO F0 mice (Numbers 1, 7, 24, and 29). (**D**) The genome sequencing map of number 29, which was used in this study as the founder for NPFFR2 KO mice. (**E**) The genomic DNA PCR products of wild-type (WT) and NPFFR2 KO mice (No. 29). (**F**) The cDNA PCR products of WT and NPFFR2 KO mice (No. 29). (**F**) The genotyping PCR products of WT and NPFFR2 KO mice (No. 29). Red arrows, indicate the specific bp of DNA markers.

NPFFR2 KO mice were generated using CRISPR/Cas9 technology. Four different sgRNAs targeting *Npffr2* were designed (Figure 1A).

After testing the gene-editing efficiency of the sgRNAs, sgRNA2 and sgRNA3 were chosen for NPFFR2 KO mice generation, because of their efficient gene editing-profiles (Figure 1B). Four NPFFR2 KO F0 mice were generated by injection of recombinant Cas9 protein and sgRNA2 or sgRNA3 into oocyte pronuclei; the animals were designated as numbers 1, 7, 24, and 29 (Figure 1C). Number 29 was further chosen as a founder for the strain used in the current study, based on its deletion profile. A 79 bp deletion in the transmembrane domain 2 of NPFFR2 protein was observed (Figure 1D). The NPFFR2 deletion was verified by performing PCR on cDNA (Figure 1E) and genomic DNA (Figure 1F) templates with a reverse primer targeting a sequence within the deleted segment. The NPFFR2 deletion was also checked by genotyping PCR, in which WT mice had a 467 bp PCR product. The PCR product for NPFFR2 KO mice exhibited a lower molecular weight than that for the WT mice (Figure 1G).

The neuroendocrine and behavioral parameters of NPFFR2 KO mice were measured. Growth curves between age 3 to 14 weeks old for male NPFFR2 KO mice showed no difference with WT control mice (Supplementary Figure S1A). The locomotor activity, sensorimotor gating, and learning behavior were also not changed in NPFFR2 KO mice (Supplementary Figure S1B–D). The depressive- and anxiety-like behaviors were not different between WT control mice and NPFFR2 KO mice (Supplementary Figure S1E–K). The physiological and behavioral parameters of female NPFFR2 KO mice were also measured. Similar to the males, growth curves between 3 and 14 weeks of age for female NPFFR2 KO mice were not different than WT control mice (Supplementary Figure S2A). The depressive- and anxiety-like behaviors were also not different between WT control mice and NPFFR2 KO mice (Supplementary Figure S2B–H).

**Figure 2:**
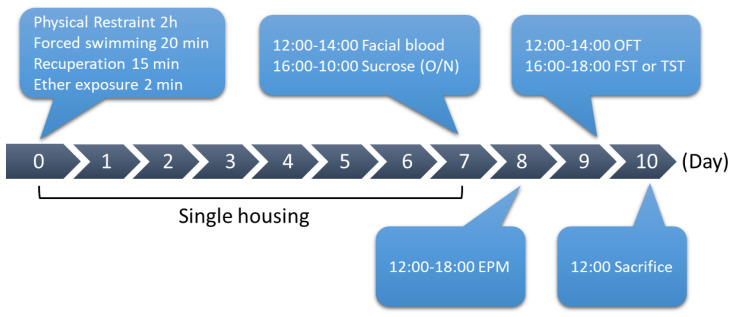
Single prolonged stress (SPS) procedure. SPS was used to induce stress response in male mice. Mice were physically restrained, subjected to forced swimming and exposed to ether on day 0. Then, the mice were singly housed for 7 days. On days 7 to 10, mice were examined in depressive- and anxiety-like behavior tests before brain tissues were collected for molecular assays. O/N, overnight.

After finding that the NPFFR2 KO mice had similar baseline characteristics to WT, SPS was used to induce stress-related behaviors in WT and NPFFR2 KO mice (Figure 2).

**Figure 3:**
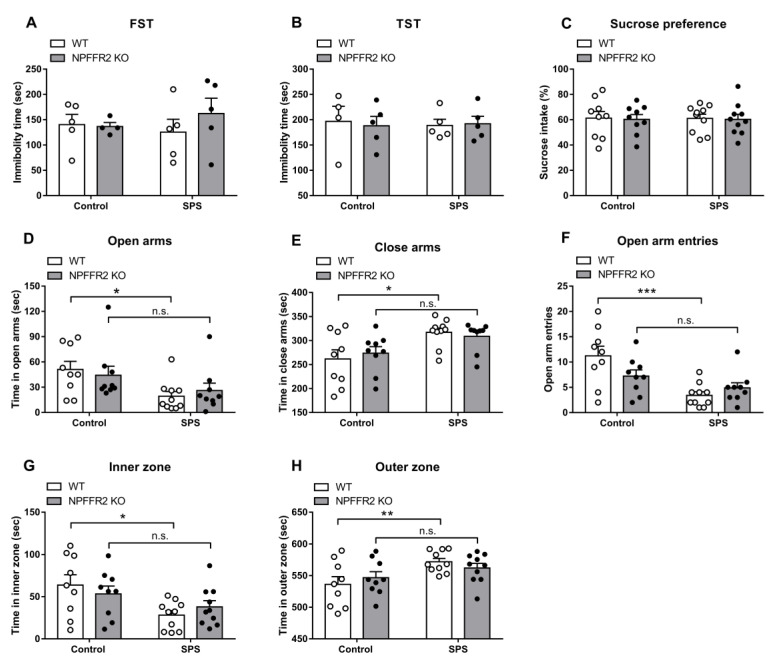
Behavioral changes in WT and NPFFR2 KO mice after SPS. Depressive- and anxiety-like behaviors were recorded for WT and NPFFR2 KO mice exposed to SPS. Depressive-like behaviors were measured by (**A**) forced swimming test (FST), (**B**) tail suspension test (TST), and (**C**) sucrose preference test. Anxiety-like behaviors were measured by elevated plus maze (EPM) and open field test (OFT). In the EPM, the durations mice spent in open arms (**D**) and closed arms (**E**), and open arm entries (**F**) were calculated. In the OFT, the durations mice spent in the inner zone (**G**) and outer zone (**H**) were calculated. Data are represented as mean ± SEM and are analyzed by two-way ANOVA with Bonferroni’s multiple comparison test. * *p* < 0.05, ** *p* < 0.01, *** *p* < 0.001, n.s., no significance; comparing control and SPS groups. FST and TST, *n* = 4–5 per group; all other tests, *n* = 8–10 per group. The white and black dots indicate the individual value of WT and NPFFR2 KO mice, respectively. EMP result of one NPFFR2 KO mouse exposed to SPS was excluded because the mouse was fallen from an open arm platform during the test.

The depressive-like behaviors were evaluated by the sucrose preference test, FST and TST. Neither WT nor NPFFR2 KO mice showed depressive-like phenotypes after exposure to SPS (Figure 3A–C).

Anxiety-like behaviors were also examined by EPM and OFT. In the EPM assay, stress exposure significantly increased the time WT mice spent in closed arms, while decreasing the time in open arms and reducing the number of open arm entries (Figure 3D–3F). Two-way ANOVA indicates significant effects of SPS exposure (open arms, F(1, 33) = 8.118, *p* = 0.0075; close arms, F(1, 33) = 11.54, *p* = 0.0018; open arm entries, F(1, 33) = 15.89, *p* = 0.0004) and interaction (open arm entries, F(1, 33) = 4.641, *p* = 0.0386). Bonferroni’s multiple comparison test reveals that SPS exposure increased the anxiety-like behaviors tested by EPM in WT mice (open arms, *p* = 0.0278; close arms, *p* = 0.0106; open arm entries, *p* = 0.0002), but not in NPFFR2 KO mice (open arms, *p* = 0.3149; close arms, *p* = 0.1514; open arm entries, *p* = 0.4198). In the OFT, WT mice with stress exposure had significantly increased time spent in the outer zone and decreased time in the inner zone (Figure 3G–H). These results indicated that SPS exposure induced anxiety-like behaviors in WT mice. On the contrary, NPFFR2 KO mice did not display anxiety-like behaviors after exposure to SPS. Two-way ANOVA indicates significant effect of SPS exposure (inner zone, F(1, 34) = 8.636, *p* = 0.0059; outer zone, F(1, 34) = 8.639, *p* = 0.0059). Bonferroni’s multiple comparison test reveals that SPS exposure increased the anxiety-like behaviors tested by OFT in WT mice (inner zone, *p* = 0.0128; outer zone, *p* = 0.0127), but not in NPFFR2 KO mice (inner zone, *p* = 0.4401; outer zone, *p* = 0.4408).

**Figure 4:**
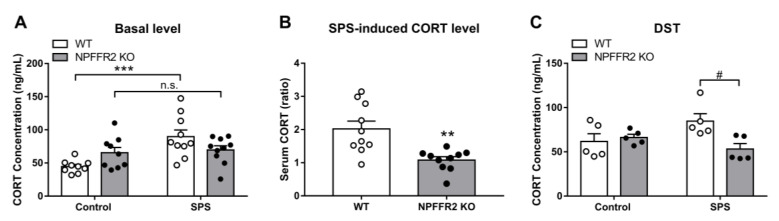
HPA axis function in WT and NPFFR2 KO mice after exposure to SPS. (**A**) The level of serum corticosterone (CORT) (WT-control, *n* = 9; WT-SPS, *n* = 10; NPFFR2-KO control, *n* = 10; NPFFR2 KO-SPS, *n* = 10). (**B**) The increase of serum CORT after mice were exposed to SPS (*n* = 10 per group). (**C**) Negative feedback on the HPA axis was tested with the dexamethasone suppression test (DST) (*n* = 5 per group). Data are represented as mean ± SEM. In Figure 4A and 4C, data are analyzed by two-way ANOVA with Bonferroni’s multiple comparison test. *** *p* < 0.001, n.s., no significance; comparing control and SPS groups; ^#^ *p* < 0.05, comparing WT and NPFFR2 KO mice. In Figure 4B, data are analyzed by unpaired Student’s *t*-test. ** *p* < 0.01, comparing WT and NPFFR2 KO mice. The white and black dots indicate the individual value of WT and NPFFR2 KO mice, respectively.

Changes in HPA axis function were also measured after mice were exposed to SPS. The exposure led to upregulation of serum CORT in WT mice, while the level of serum CORT did not change in NPFFR2 KO mice exposed to SPS (Figure 4A). Two-way ANOVA indicates significant effects of SPS exposure (F(1, 34) = 11, *p* = 0.0022) and interaction (F(1, 34) = 7.613, *p* = 0.0093). Bonferroni’s multiple comparison test reveals that SPS exposure increased the level of serum CORT in WT mice (*p* = 0.0003) but not in NPFFR2 KO mice (*p* > 0.9999).

Correspondingly, the SPS-induced serum CORT increase was significantly reduced in NPFFR2 KO mice compared to WT mice (Figure 4B). The unpaired Student’s *t*-test indicates a decrease in NPFFR2 KO mice compared to WT mice (*p* = 0.0018). Negative feedback on the HPA axis was then tested with the DST. The effect of dexamethasone on the reduction of serum CORT level was validated in WT mice (Supplementary Figure S3). After mice were injected with dexamethasone and physically restrained for 10 min, SPS-exposed WT mice tended to show higher serum CORT levels, but the difference with unexposed control mice was not statistically significant. Importantly, the level of serum CORT in NPFFR2 KO mice was significantly lower than that in WT mice after SPS exposure (Figure 4C). Two-way ANOVA indicates significant effect of interaction between SPS exposure and mice genotypes (F(1, 16) = 6.642, *p* = 0.0203). Bonferroni’s multiple comparison test reveals that SPS exposure tended to increase the serum CORT in WT mice (*p* = 0.0674), and the levels of serum CORT were reduced in NPFFR2 KO mice compared to WT mice (*p* = 0.0115).

**Figure 5:**
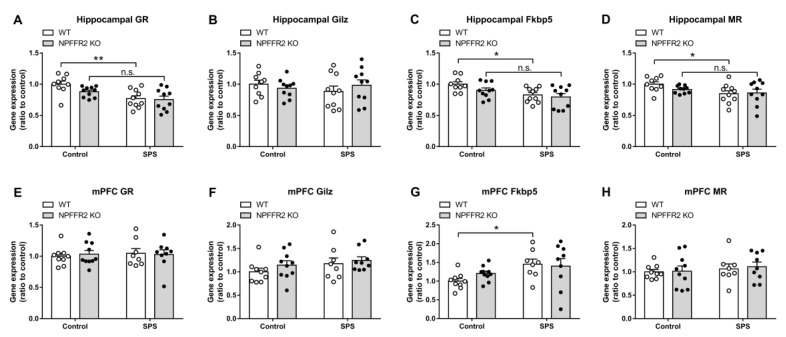
mRNA levels of glucocorticoid receptor (GR), GR-regulated genes and mineralocorticoid receptor (MR) in hippocampus and medial prefrontal cortex (mPFC) after SPS exposure. The hippocampus and mPFC of WT and NPFFR2 KO mice were collected from control and SPS-exposed mice, then analyzed for expression of the designated genes. (**A**) Hippocampal GR, (**B**) Hippocampal GC-Induced-Leucine-Zipper (*Gilz*), (**C**) Hippocampal FK506-binding protein 5 (*Fkbp5*), and (**D**) Hippocampal MR (WT-control, *n* = 9; WT-SPS, *n* = 10; NPFFR2-KO control, *n* = 10; NPFFR2 KO-SPS, *n* = 10). (**E**) mPFC GR, (**F**) mPFC *Gilz*, (**G**) mPFC *Fkbp5* and (**H**) mPFC MR (WT-control, *n* = 9; WT-SPS, *n* = 10; NPFFR2-KO control, *n* = 8; NPFFR2 KO-SPS, *n* = 9.). Data are represented as mean ± SEM and are analyzed by two-way ANOVA with Bonferroni’s multiple comparison test. * *p* < 0.05, ** *p* < 0.01, n.s., no significance; comparing control and SPS groups. The white and black dots indicate the individual value of WT and NPFFR2 KO mice, respectively.

The gene expression levels for GR and GR-regulated genes, GC-Induced-Leucine-Zipper (*Gilz*), and FK506-binding protein 5 (*Fkbp5*), as well as MR were measured in the hippocampus and medial prefrontal cortex (mPFC) by real-time PCR. The hippocampal GR and *Fkbp5* expression levels were reduced in WT mice after exposure to SPS, with no change of *Gilz* mRNA. The expression of hippocampal MR was also decreased in WT mice after exposure to stress. However, the SPS exposure did not alter the expression levels of those genes in NPFFR2 KO mice (Figure 5A–5D). Two-way ANOVA indicates significant effect of SPS exposure (GR, F(1, 35) = 15.09, *p* = 0.0004; *Fkbp5*, F(1, 35) = 9.834, *p* = 0.0035; MR, F(1, 35) = 5.441, *p* = 0.0255). Bonferroni’s multiple comparison test reveals that SPS exposure reduced the gene expression in WT mice (GR, *p* = 0.0024; *Fkbp5*, *p* = 0.0202; MR, *p* = 0.0432), but not in NPFFR2 KO mice (GR, *p* = 0.1182; *Fkbp5*, *p* = 0.1957; MR, *p* = 0.7768).

Moreover, SPS exposure does not change most of the mRNA levels of GR, GR-regulated genes or MR in the mPFC of either WT or NPFFR2 KO mice (Figure 5E, F and H). Only the *Fkbp5* increased after WT mice exposed to SPS (Figure 5G). Two-way ANOVA indicates significant effect of SPS exposure (*Fkbp5*, F(1, 32) = 6.483, *p* = 0.0159). Bonferroni’s multiple comparison test reveals that SPS exposure increased the *Fkbp5* expression in WT mice (*p* = 0.0408).

**Figure 6:**
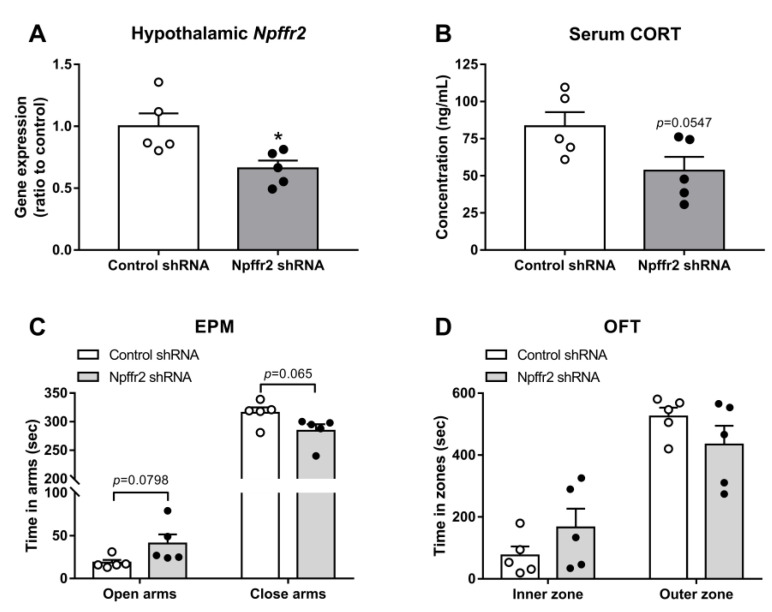
Intra-hypothalamic paraventricular nucleus (PVN) *Npffr2* silencing affects behavioral and biochemical endpoints in mice exposed to SPS. Mice were exposed to SPS 1–2 weeks after intra-PVN lenti-*Npffr2-*shRNA injection. (**A**) The effect of hypothalamic *Npffr2* gene knockdown. (**B**) The level of serum CORT after SPS exposure. Anxiety-like behaviors were measured by (**C**) EPM and (**D**) OFT. Data are represented as mean ± SEM and are analyzed by unpaired Student’s *t*-test. * *p* < 0.05; comparing control and *Npffr2*-shRNA treatment groups (*n* = 5 per group). The white and black dots indicate the individual value of WT and NPFFR2 KO mice, respectively.

The hypothalamic PVN controls the activity of the HPA axis. To test whether PVN NPFFR2 is involved in the SPS-induced behavioral changes seen in WT mice, the mice were bilaterally injected in the PVN with lenti-*Npffr2*-shRNA. The gene silencing effect was previously verified in the olfactory bulb [15] and confirmed by real-time PCR in the current study (Figure 6A). The unpaired Student’s *t*-test indicates a reduction in NPFFR2 mRNA in *Npffr2*-shRNA group compared to control-shRNA group (*p* = 0.0236).

After mice were exposed to SPS, the level of serum CORT showed a trend toward decrease in the *Npffr2*-shRNA-treated group compared to controls (*p* = 0.0547, unpaired Student’s *t*-test) (Figure 6B). *Npffr2*-shRNA-treated mice also showed a trend toward less anxiety-like behavior in the EPM (*p* = 0.0798, time in open arms; *p* = 0.065, time in closed arms, unpaired Student’s *t*-test) (Figure 6C). Similar behavioral phenomena were observed in OFT, with a trend toward lower anxiety-like behavior but no significant difference between groups (Figure 6D).

## 4. Discussion

In the current study, we generated a NPFFR2 congenital KO mice with CRISPR/Cas9 technology and examined the impact of NPFFR2 deletion on stress responses elicited by SPS. The findings were also verified by intra-PVN *Npffr2* silencing in WT mice. Under basal conditions, the NPFFR2 KO mice did not exhibit any apparent behavioral difference with WT mice, including locomotor activity, cognition-associated behaviors or emotional responses similar to anxiety and depression. After WT mice were exposed to SPS, the animals displayed clear anxiety-like behaviors (evidenced by EMP and OFT) with no depressive-like behaviors (according to FST, TST, and sucrose preference test). Interestingly, NPFFR2 deletion in mice caused the animals to be resistant to SPS exposure; hence, the KO mice did not exhibit any behavioral changes. The results of our experiments measuring HPA axis function were consistent with the behavioral outcomes. As such, SPS exposure elevated serum CORT in WT mice but not NPFFR2 KO mice. Negative feedback on the HPA axis was also tested by injecting dexamethasone to the mice. NPFFR2 KO mice displayed a sensitive negative feedback response compared to WT mice after exposure to SPS. SPS reduced the hippocampal expression of GR, a GR co-chaperone protein (FKBP5), and MR in WT mice, but no gene expression differences were observed in NPFFR2 KO mice. Notably, the expression levels of most genes were not changed in the mPFC of SPS-exposed WT or NPFFR2 KO mice, except FKBP5 which was increased in SPS-exposed WT mice. To test the impact of hypothalamic NPFFR2 on SPS response, PVN *Npffr2* was knocked down by intra-PVN injection of *Npffr2*-shRNA in WT mice. Mice receiving control-shRNA and *Npffr2*-shRNA were exposed to SPS, and HPA axis function was reduced in mice treated with *Npffr2*-shRNA, according to the level of serum CORT. Mice treated with *Npffr2*-shRNA also tended to show reduced anxiety-like behaviors compared to control mice. Together, our findings suggest that NPFFR2 deletion attenuates SPS-induced stress responses in both behavioral and biochemical aspects, and the effects are partially mediated by NPFFR2 in the hypothalamic PVN.

Chronic stress and high sustained levels of circulating CORT frequently underlie mood disorders, including depression and anxiety [17]. Many reports have shown that RF-amine peptides regulate the activity of the HPA axis; such RF-amine peptides include: NPFF, prolactin releasing peptide, neuropeptide SF, neuropeptide AF, and kisspeptin-13 [10,11]. However, the impact of NPFF has only begun to be explored in recent years. NPFFR2 has been demonstrated to directly activate the HPA axis through the hypothalamic PVN, triggering anxiety-like behaviors [12,14]. In the current study, we verified the impact of NPFFR2 knockdown on stress-related disorders. SPS is often used as a model of PTSD, with aberrant HPA axis activity but different from chronic stress [18]. After exposure to SPS, rodents will display anxiety-like behaviors, increased fear responses, reduced social interaction, and impaired learning and memory [18]. In our study, WT mice exposed to SPS exhibited anxiety-like behaviors, including decreased time spent in open arms of the EPM and in the inner zone of the OFT, without the occurrence of depressive-like phenotypes. Interestingly, the NPFFR2 KO mice were resistant to the effects of SPS exposure, and the SPS-exposed KOs exhibited normal behaviors. These findings suggest that NPFFR2 is crucial to SPS-induced anxiogenic responses. These results are consistent with previous findings, which showed that chronic activation of NPFFR2 in mice induces clear anxiety- and depressive-like behaviors [15]. WT mice exposed to SPS also had increased circulating CORT. Similar to behavioral responses, serum CORT of NPFFR2 KO mice was not elevated after exposure to SPS. Our current results suggest that without the influence of NPFFR2, SPS cannot mediate activation of the HPA axis. NPFFR2 have been reported to regulate the diet-induced adaptive thermogenesis via a hypothalamic NPY-dependent pathway which might affect the stress responses [8]. However, this impact is less likely to happen in the current study since we did not observe the body weight change during the monitoring of growth curve.

Chronic stress and HPA axis hyperactivity also impair hippocampal function, leading to reduced hippocampal GR protein and disruption of HPA axis negative feedback [15,17]. Sustained high levels of circulating CORT are thought to target hippocampal GR and MR, which might also lead to hippocampal atrophy [19]. Although in the current study, SPS did not significantly impair negative feedback of the HPA axis (*p* = 0.0917) in WT mice, those mice with knockdown of NPFFR2 appeared to have sensitive negative feedback after exposure to SPS. These data align with our findings that hippocampal GR and FKBP5 were reduced in WT mice after SPS exposure but not in NPFFR2 KO mice. Hippocampal GR was shown to be reduced in depression patients [20], and our result showed reduced hippocampal GR in WT mice which reflects this clinical finding. This effect might be due to long-term elevation of circulating CORT triggered by SPS. The lower expression of hippocampal GR in WT mice might also impair the hippocampus-mediated negative feedback on the HPA, which could otherwise enhance SPS-induced HPA axis hyperactivity.

In our study, SPS exposure caused a chronic stress response, but it did not resemble a model of PTSD. In a true PTSD model, the stressor should decrease circulating CORT and enhance the glucocorticoid negative feedback via upregulated hippocampal GR [21]. Seven-days of SPS was previously shown to increase GR mRNA but decrease MR mRNA in hippocampal CA1, with a reduction of MR/GR ratio [22]. However, we observed a decrease of both hippocampal GR and MR mRNA levels, which corresponded to reduced negative feedback in our stress model. The different responses in chronic stress and PTSD models might reflect the influence of habituation and sensitization of the HPA axis [23]. Apparently, our SPS protocol caused signs of habituation to glucocorticoid activity with unknown reason(s), possibly because of the animal facility environment, animal species or strain. Homeostasis and modulation of the HPA axis are complex processes that can be influenced by a wide variety of different physiological parameters. The unique phenotype related to the HPA axis in PTSD patients has been difficult for researchers to recapitulate in PTSD animal models [24]. Nevertheless, our findings are supported by previous reports that NPFFR2 signals are important to stress-induced HPA axis hyperactivity and emotional responses [14,15].

NPFFR2 was reported to activate the HPA axis through increased PVN neuronal activity [14]. The knockdown of PVN NPFFR2 was also shown to ameliorate chronic mild stress-induced depressive-like behaviors [15]. Thus, we verified the role of PVN NPFFR2 in SPS-induced anxiogenic behavior changes. After injection of Lenti-intra-PVN *Npffr2* shRNA, WT mice were then subjected to SPS. In our study, PVN NPFFR2 knockdown did not fully attenuate the impact of SPS exposure. However, we did observe similar effects of PVN *Npffr2* silencing on anxiety-like behaviors and HPA axis function between PVN NPFFR2 knockdown mice and congenital NPFFR2 KO mice. The findings suggest that different brain areas, in addition to the PVN, might play a role(s) in SPS-induced responses. Indeed, it is plausible that NPFFR2 in the hippocampus may play an important role in SPS, because of hippocampal mediation of negative feedback on the HPA axis.

In summary, our findings reveal that ablation of NPFFR2 from mice attenuates the behavioral and molecular responses of mice exposed to SPS. NPFFR2 deletion diminished SPS-induced HPA axis hyperactivity and GR downregulation, and it impaired negative feedback of the HPA and lessened anxiety-like behaviors. Thus, NPFFR2 could serve as a potential therapeutic target for stress-related disorders.

## Supplementary Materials

The following are available online at https://www.mdpi.com/2073-4409/9/11/2479/s1, Figure S1: The behavioral profiles of male NPFFR2 KO mice. Figure S2: The behavioral profiles of female NPFFR2 KO mice. Figure S3: The dexamethasone suppression test in male WT mice.

## References

[1] Cohen S., Janicki-Deverts D., Miller G.E. (2007). Psychological Stress and Disease. JAMA.

[2] Ulrich-Lai Y.M., Herman J.P. (2009). Neural regulation of endocrine and autonomic stress responses. Nat. Rev. Neurosci..

[3] Bao A.-M., Swaab D.F. (2019). The human hypothalamus in mood disorders: The HPA axis in the center. Ibro Rep..

[4] Pittenger C., Duman R.S. (2008). Stress, Depression, and Neuroplasticity: A Convergence of Mechanisms. Neuropsychopharmacology.

[5] De Kloet E.R., Joëls M., Holsboer F. (2005). Stress and the brain: From adaptation to disease. Nat. Rev. Neurosci..

[6] Panula P., Aarnisalo A.A., Wasowicz K. (1996). Neuropeptide FF, a mammalian neuropeptide with multiple functions. Prog. Neurobiol..

[7] Lin Y.-T., Liu H.-L., Day Y.-J., Chang C.-C., Hsu P.-H., Chen J.-C. (2017). Activation of NPFFR2 leads to hyperalgesia through the spinal inflammatory mediator CGRP in mice. Exp. Neurol..

[8] Zhang L., Ip C.K., Lee I.-C.J., Qi Y., Reed F., Karl T., Low J.K., Enriquez R.F., Lee N.J., Baldock P.A. (2018). Diet-induced adaptive thermogenesis requires neuropeptide FF receptor-2 signalling. Nat. Commun..

[9] Lin Y.-T., Yu Z., Tsai S.-C., Hsu P.-H., Chen J.-C. (2020). Neuropeptide FF receptor 2 inhibits capsaicin-induced CGRP Upregulation in mouse trigeminal ganglion. J. Headache Pain.

[10] Chen J.-C., Lin Y.-T. (2019). Neuropeptide FF modulates neuroendocrine and energy homeostasis through hypothalamic signaling. Chin. J. Physiol..

[11] Jhamandas J.H., Goncharuk V. (2013). Role of neuropeptide FF in central cardiovascular and neuroendocrine regulation. Front. Endocrinol..

[12] Jhamandas J.H., Simonin F., Bourguignon J.-J., Harris K.H. (2007). Neuropeptide FF and neuropeptide VF inhibit GABAergic neurotransmission in parvocellular neurons of the rat hypothalamic paraventricular nucleus. Am. J. Physiol. Integr. Comp. Physiol..

[13] Gouardères C., Faura C., Zajac J.-M. (2004). Rodent strain differences in the NPFF1 and NPFF2 receptor distribution and density in the central nervous system. Brain Res..

[14] Lin Y.-T., Yu Y.-L., Hong W.-C., Yeh T.-S., Chen T.-C., Chen J.-C. (2017). NPFFR2 Activates the HPA Axis and Induces Anxiogenic Effects in Rodents. Int. J. Mol. Sci..

[15] Lin Y.-T., Liu T.-Y., Yang C.-Y., Yu Y.-L., Chen T.-C., Day Y.-J., Chang C.-C., Huang G.-J., Chen J.-C. (2016). Chronic activation of NPFFR2 stimulates the stress-related depressive behaviors through HPA axis modulation. Psychoneuroendocrinology.

[16] Paxinos G., Franklin K.B.J. (2001). The Mouse Brain in Stereotaxic Coordinates.

[17] Nestler E.J., Barrot M., Dileone R.J., Eisch A.J., Gold S.J., Monteggia L.M. (2002). Neurobiology of Depression. Neuron.

[18] Souza R.R., Noble L.J., McIntyre C.K. (2017). Using the Single Prolonged Stress Model to Examine the Pathophysiology of PTSD. Front. Pharmacol..

[19] Holsboer F. (2000). The Corticosteroid Receptor Hypothesis of Depression. Neuropsychopharmacology.

[20] Alt S.R., Turner J.D., Klok M.D., Meijer O.C., Lakke E.A., DeRijk R.H., Muller C.P. (2010). Differential expression of glucocorticoid receptor transcripts in major depressive disorder is not epigenetically programmed. Psychoneuroendocrinology.

[21] Yamamoto S., Morinobu S., Takei S., Fuchikami M., Matsuki A., Yamawaki S., Liberzon I. (2009). Single prolonged stress: Toward an animal model of posttraumatic stress disorder. Depress. Anxiety.

[22] Liberzon I., López J.F., Flagel S.B., Vázquez D.M., A Young E. (1999). Differential Regulation of Hippocampal Glucocorticoid Receptors mRNA and Fast Feedback: Relevance to Post-Traumatic Stress Disorder. J. Neuroendocr..

[23] Pitman D.L., Ottenweller J.E., Natelson B.H. (1990). Effect of stressor intensity on habituation and sensitization of glucocorticoid responses in rats. Behav. Neurosci..

[24] Liberzon I., Krstov M., Young E.A. (1997). Stress-restress: Effects on ACTH and fast feedback. Psychoneuroendocrinology.

