# Supplementary material for: Ablation of NPFFR2 in Mice Reduces Response to Single Prolonged Stress Model
Source: Cells. 2020 Nov 14;9(11):2479. doi: 10.3390/cells9112479 (PMC7697606; doi:10.3390/cells9112479)
Supplement: Supplementary file 1 [file cells-09-02479-s001.pdf]

Supplementary Figures

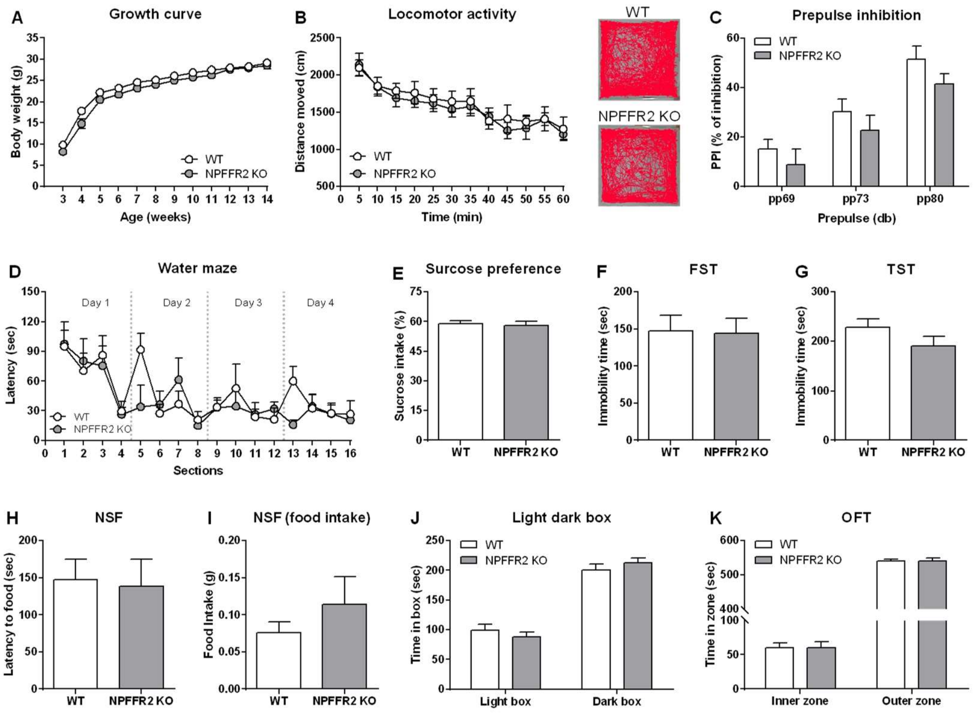

Supplementary Figure S1. The behavioral profiles of male NPFFR2 knockout (KO) mice. (**A**) Growth curves of mice from 3 weeks of age to week 14 (wild-type (WT), *n* = 11; KO, *n* = 7). (**B**) Locomotor activity within 60 min (WT, *n* = 9; KO, *n* = 10). (**C**) Sensorimotor gating was tested by prepulse inhibition (WT, *n* = 9; KO, *n* = 10). (**D**) The water maze was tested for learning (WT, *n* = 4; KO, *n* = 5). The depressive-like behaviors were tested by (**E**) sucrose preference test (WT, *n* = 9; KO, *n* = 10), (**F**) forced swimming test (FST) (WT, *n* = 9; KO, *n* = 10), and (**G**) tail suspension test (TST) (WT, *n* = 9; KO, *n* = 10). Anxiety-like behaviors were tested by (**H** and **I**) novelty-suppressed feeding (NSF) (WT, *n* = 9; KO, *n* = 10), (**J**) light-dark box (WT, *n* = 9; KO, *n* = 10), and (**K**) open field test (OFT) (WT, *n* = 9; KO, *n* = 10). Data are presented as mean ± SEM. The data in S1E–I are analyzed by the unpaired Student's *t*-test. The data in S1A–D, S1J, S1K are analyzed by two-way ANOVA with Bonferroni's multiple comparison test.

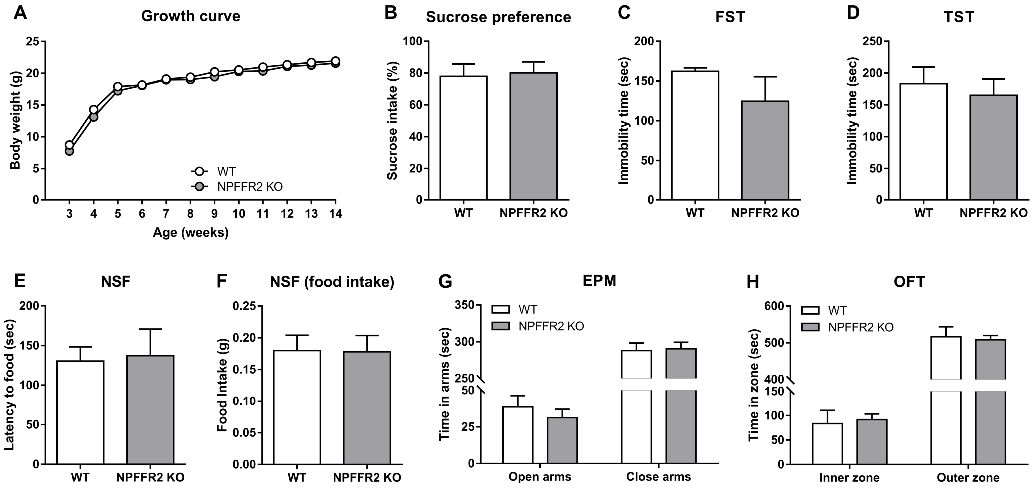

Supplementary Figure S2. The behavioral profiles of female NPFFR2 KO mice. (**A**) Growth curves of mice from 3 weeks of age to week 14. The depressive-like behaviors were tested by sucrose preference (**B**), FST (**C**), and TST (**D**). The anxiety-like behaviors were tested by NSF (**E** and **F**), Elevated plus maze (EPM) (**G**), and OFT (**H**). Data are presented as mean ± SEM (WT, $n = 5$; KO, $n = 5$). The data in S2B–F are analyzed by the unpaired Student's *t*-test. The data in S2A, S2G, S2H are analyzed by two-way ANOVA with Bonferroni's multiple comparison test.

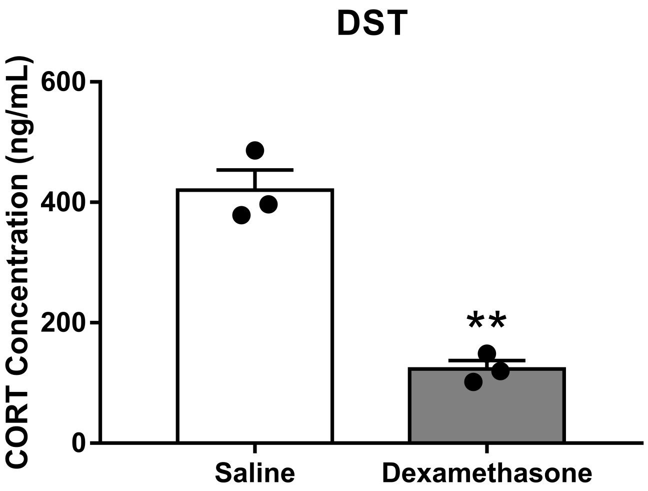

Supplementary Figure S3. The dexamethasone suppression test in male WT mice. A pioneer test was performed to validate the effect of dexamethasone on the reduction of circulating CORT levels. WT mice were i.p. injected with saline or 0.1 mg/kg dexamethasone 1 h before mice were physically restrained for 10 min. Facial blood was collected immediately for CORT ELISA assay. Data are presented as mean ± SEM and are analyzed by the unpaired Student's *t*-test. ** $p < 0.01$, comparing saline to dexamethasone group ($n = 3$ per group). The black dots indicate the individual value of WT mice.
